# The effectiveness of modern cardiac rehabilitation: A systematic review of recent observational studies in non-attenders versus attenders

**DOI:** 10.1371/journal.pone.0177658

**Published:** 2017-05-12

**Authors:** Jennifer Sumner, Alexander Harrison, Patrick Doherty

**Affiliations:** University of York, Department of Health Sciences, York, United Kingdom; University of Illinois at Urbana-Champaign, UNITED STATES

## Abstract

**Background:**

The beneficial effects of cardiac rehabilitation (CR) have been challenged in recent years and there is now a need to investigate whether current CR programmes, delivered in the context of modern cardiology, still benefit patients.

**Methods:**

A systematic review of non-randomised controlled studies was conducted. Electronic searches of Medline, Embase, CINAHL, science citation index (web of science), CIRRIE and Open Grey were undertaken. Non-randomised studies investigating the effects of CR were included when recruitment occurred from the year 2000 onwards in accordance with significant CR guidance changes from the late 1990’s. Adult patients diagnosed with acute myocardial infarction (AMI) were included. Non-English articles were considered. Two reviewers independently screened articles according to pre-defined selection criteria as reported in the PROSPERO database (CRD42015024021).

**Results:**

Out of 2,656 articles, 8 studies involving 9,836 AMI patients were included. Studies were conducted in 6 countries. CR was found to reduce the risk of all-cause and cardiac-related mortality and improve Health-Related Quality of Life (HRQOL) significantly in at least one domain. The benefits of CR in terms of recurrent MI were inconsistent and no significant effects were found regarding re-vascularisation or re-hospitalisation following AMI.

**Conclusion:**

Recent observational evidence draws different conclusions to the most current reviews of trial data with respect to total mortality and re-hospitalisation, questioning the representativeness of historic data in the modern cardiological era. Future work should seek to clarify which patient and service level factors determine the likelihood of achieving improved all-cause and cardiac mortality and reduced hospital re-admissions.

## Introduction

Coronary heart disease (CHD) is a huge global problem accounting for the leading cause of death worldwide [1]. Acute myocardial Infraction (AMI) is the most common cause of death from CHD and is associated with 188, 000 hospital episodes each year in the United Kingdom (UK) alone, representing a major cause of death and ill health [2]. Recognising this burden and the need to rehabilitate patients National, European and International guidelines recommend the provision of cardiac rehabilitation (CR) services [3–6]. In brief, CR is a multi-component intervention generally comprising of structured exercise training, psychological support and education to promote positive lifestyle changes. Improvements in risk factors, mortality, morbidity and Health-Related Quality of Life (HRQOL) have all been associated with CR attendance [4, 7–9].

Despite the many documented advantages associated with CR, utilisation is highly variable and relatively low [10–12]. Across Europe an estimated 2 million eligible patients per year access CR but less than 40% uptake CR [13]. Comparatively in 2016 the National Audit of Cardiac Rehabilitation (NACR) for England, Wales and Northern Ireland reported an overall uptake to CR of 50%, placing the UK in the top 2% of countries for uptake in Europe, but there are still improvements to be made [11].

In recent years research has focused on innovations to improve referral and uptake [14]. However, the benefits of CR, as delivered in the context of the present day, have been challenged. The largest pragmatic Randomised Controlled Trial (RCT) of modern day CR in the UK; the RAMIT trial, found no significant beneficial effects on mortality, cardiac or psychological morbidity, risk factors, HRQOL or activity level from CR [15]. Although methodological issues in this trial led to questions around the validity of the study findings [16], an important question around CR efficacy was raised. Since the RAMIT RCT the most recent 2016 Cochrane review on CR effectiveness identified no current RCTs which have been conducted with sufficient sample sizes to investigate efficacy [9]. Given the practical and ethical challenges, as CR is standard care, it seems improbable any such trial could occur. But the question remains with improvements in patient treatment, increasingly diverse programme components and changes to the profile of patients receiving CR today versus historic counterparts does modern day CR still benefit patients [17–20]. In an effort to overcome the aforementioned challenges, extend the external validity of trials and determine the benefit of current day CR in routine practice a recently published systematic review of RCTs and non-randomised studies investigated efficacy in the post-statin era in a mixed CR population [21]. The primary outcome; total mortality following CR, was confirmed although the secondary outcomes of cardiac mortality and re-hospitalisation were not evident contrary to the most recent Cochrane review of RCT evidence [9, 21]. Conversely another RCT review of CR in post-MI patients concluded a reduction in both all-cause and cardiac mortality as well as re-hospitalisation [22].

To further understanding on the effects of modern day CR in routine practice this systematic review will investigate a more specific homogenous CR population (AMI patients) and extend the outcomes considered by the CROS review. Specifically, recent observational studies investigating the effect of CR versus no rehab in AMI patients alone (with or without revascularisation) considering HRQOL outcomes in addition to mortality, hospital re-admission, re-occurrence of AMI and re-vascularisation. CR programme format and the intervention components used will also be reviewed.

## Methods

The study is reported and conducted according to the Preferred Reporting Items for Systematic reviews and Meta-Analyses (PRISMA) guidelines [23] and Guidelines for the Meta-Analyses and Systematic Reviews of Observational Studies (MOOSE) [24]. The systematic review protocol was prospectively registered on the PROPSPERO database of systematic reviews (registration number: CRD42015024021). A copy of the PRISMA checklist is included in the supplements (S1 Table).

### Literature search

Medline, Embase, CINAHL, science citation index (web of science), CIRRIE and Open Grey were electronically searched for relevant articles. Combinations of medical subject headings and keywords around the following themes were used; cardiac population descriptors, CR intervention, CR use, patient outcomes. The search strategy was developed in conjunction with a trained information specialist and conducted in June 2015. An updated search was run in November 2016 to identify any further articles published since the initial search. The reference lists of included studies were also hand searched for further relevant studies. A copy of the Medline search strategy is included in the supplements (S2 Table).

### Study selection

Titles and abstracts of identified citations were screened for inclusion by a single reviewer. Potentially eligible articles were then full text screened independently by two reviewers according to the inclusion criteria. Disagreements regarding eligibility were discussed and resolved by a third reviewer. In instances of unclear reporting authors were contacted to provide further information and clarity. The eligibility criteria are described as follows:

**Participants:** Male or female adults diagnosed with AMI; either ST-elevated (STEMI) or non-ST-elevated (nSTEMI) were included. Both medically managed (i.e. drug therapies) or re-vascularised (Coronary Artery Bypass Graft Surgery, or Percutaneous Coronary Intervention) AMI patients were included. The AMI population was chosen as the predominant cause of CHD related death and to minimise heterogeneity in the analyses population i.e. by factoring the impact of different care pathways.

**Intervention:** CR delivered as a structured, multi-component programme which included exercise and/or structured physical activity in addition to at least one of the following: information provision, education, health behaviour change, psychological support or intervention and social support. CR programmes using a mixture of supervised or unsupervised approaches conducted in any setting (inpatient, outpatient, community, home based) were included.

**Control:** Patients, as defined previously, who did not participate in CR. It was anticipated that patients in the control group were only medically supervised, usually by a general practitioner or equivalent, but may have also attended unstructured prevention programmes.

**Study type:** Observational studies (prospective or retrospective cohort, case-control data from routine practice) comparing CR attenders to non-attenders were included.

**Primary outcome:** All cause- and cardiac-related mortality. Secondary outcomes included all cause and cardiac-related hospital re-admission, re-occurrence of AMI, re-vascularisation and HRQOL.

**Other criteria:** As a review of CR practice in the current day the search strategy and population inclusion was date limited. In 2000 the National Service Framework for coronary heart disease was published in the UK, detailing modern standards of care, including CR services [25]. The American Heart Association published position statements on CR programmes and CR core components in 1994 [26] and 2000 [27], a position paper by the European Society of Cardiology in 2003 provided recommendations on the design and development of CR programmes [28] and in 2001 Cochrane published the first review to define exercise based CR [29]. In line with the establishment of international modern standards of care in CR the search strategy was restricted to publications from 2000 to present day. The populations within identified studies were then screened according to their recruitment date and excluded if pre-2000. Foreign language papers were included and translated where possible.

### Data extraction

Data extraction was undertaken by one reviewer and independently checked for quality and accuracy by a second reviewer. Data items including study and population characteristics, intervention details, outcome measures and methods used to adjust for confounding were extracted. For each CR programme the components which formed the programme were identified and coded i.e. education, dietary advice etc. Adjusted effect outcomes were extracted for analyses where available. Data was extracted closest to one year follow-up. In instances where multiple adjusted outcome estimates were provided the following rules were used to decide which adjusted estimate was used in the meta-analyses: the estimate which adjusts for the maximum number of covariates, the estimate which is identified as the primary adjusted model, the estimate which includes the largest number of confounders considered important from the outset.

When multiple publications were identified for one study the primary study publication was extracted and the additional publications were searched for additional information. The extraction sheet was piloted on a sample of papers and refinements made prior to full data extraction.

### Quality assessment

Individual observational studies were assessed for quality according to the checklist developed by Wells and colleagues [30]. In brief the checklist assesses study design, confounding, selective reporting and directness. The checklist was adapted for the purposes of this study. The quality assessment questions included were as follows: 1)Was there a relevant comparison group? 2) How were the groups formed? 3) Were the comparability of groups assessed by potential confounders? 4) Did the researchers describe how potential confounding domains were decided? 5) Did the researchers consider the following potential confounders: age, gender, ethnicity, SES, region, previous event, comorbidities? 6) Did the researchers control for confounding through matching at the enrolment stage or in the analysis (adjustment)? 7) Is there evidence that specified confounders did not cause confounding? 8) Did the analysis control for confounding with adequate care? 9) Is there evidence that the study cohort was selected from a larger cohort for which data was available? 10) Is there evidence of multiple adjusted analyses conducted but only one reported? 11) Were subgroups defined in unusual ways and statistically significant results reported? 12) Is there evidence of multiple methods being used for missing data and only one approach selectively chosen and reported? 13) Is there evidence of outcomes being converted to categorical data with unusual cut off points?. Observational studies are more prone to bias than RCTs and as such it was critical that an exploration of planned adjustment for confounders was conducted [31]. Variables which studies may have considered include: age, gender, ethnicity, socioeconomic status, region, previous cardiac event and presence of comorbidities [32–34].

### Data synthesis

In order to pool data where the same outcome is reported in different formats a generic inverse variance method was used to generate an overall effect estimate for each outcome. A random effects model was used to account for study heterogeneity. Effect outcomes were reported as odds ratios (OR) with 95% confidence intervals (CI). Where meta-analysis was not possible a narrative synthesis was generated. Adjusted and unadjusted effect outcomes were presented in separate sub groups to account for the differing level of bias in each. It is well known that heterogeneity is often higher in systematic reviews of non-randomised studies [31]. Heterogeneity was evaluated through visual examination of the forest plots and the I^2^ statistic. ‘Low’ heterogeneity was set at ≤25%, ‘moderate’ 50% and ‘high’ 75% [35].

## Results

**Fig 1:**
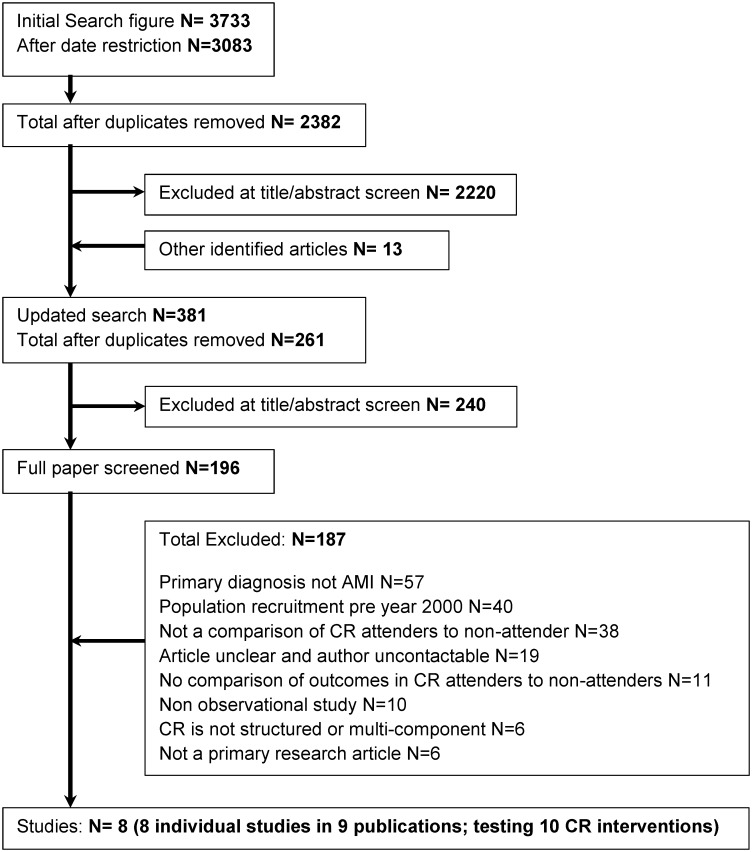
Prisma flow diagram.

A total of 3,733 articles were identified from the initial search strategy, reducing to 2,382 after duplicates and date restrictions were applied. A further 13 articles were identified from author contacts and 261 articles from an updated literature run in November 2016. Full text screening was conducted on 196 articles, according to the eligibility criteria, which resulted in 8 included studies testing 10 CR interventions (Fig 1).

### Quality assessment

**Fig 2:**
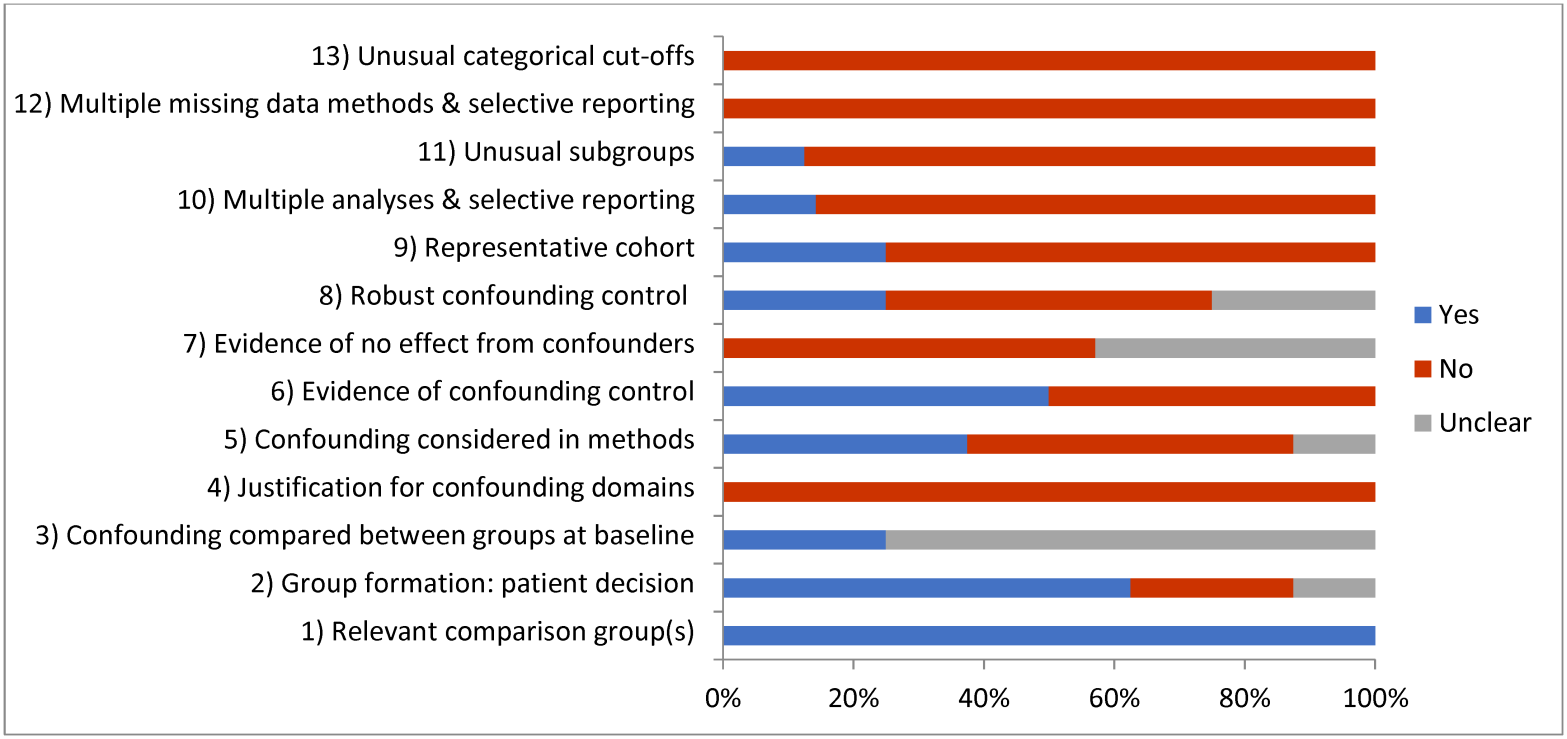
Quality appraisal.

Results of the quality assessment are presented in Fig 2. No study protocols were identified for the included studies, as such all questions relating to pre-publication of a protocol could not be considered and were removed. Most studies did not consider confounders i.e. analyses conducted without adjustment for socio-demographic background. However, for the majority of papers unusual cut-offs or subgroups and selective reporting of analyses or findings were not evident. All studies used appropriate comparison groups; either formed through patient choice or physician decision. One study used a historic control which may have introduced bias [36].

### Study characteristics

Characteristics of the included studies are presented in Table 1. The included studies comprised a total sample of 9,836 AMI patients typically followed up over 1 year. Patients were predominantly male in all studies and the age ranged from 49.9–70.0 years. Studies were conducted in a number of countries; Germany (n = 2), Spain (n = 2), United States of America (USA) (n = 1), Canada (n = 1), Korea (n = 1) and Denmark (n = 1). In terms of the CR intervention programmes most frequently paired exercise with an education component (n = 5) and typically included ≥3 intervention components (n = 8). European based studies tended to include a greater number of components in their CR programmes compared to the American and Canadian counterparts. Most used a health centre or clinic to deliver their interventions (n = 9) and included a group based approach (n = 7). Half of the interventions reported the involvement of a multi-disciplinary team the remaining studies did not report sufficiently to ascertain this point, although all programmes were described as multi-component.

**Table 1:** Study characteristics.

Author, year	Country	Intervention Group N, Mean age (SD), Gender (% male)	Intervention components	Control Group N, Mean age (SD), Gender (% male)	Follow-up period	Inpatient/ Outpatient & Setting	Individual or group approach	Multi-disciplinary team involved
Aldana S 03 & 06, Ornish CR programme[38, 40]	USA	N = 28, 56.6 years (SD 9.4), 85% male	Exercise, stress management, support group	N = 28, 58.7 years (SD 12.5), 89% male	3 & 6 months	Unclear, Healthcare centre	Both	Yes
Traditional CR	USA	N = 28, 59.9 years (SD 11.9), 71% male	Exercise, education	As above	As above	Unclear, Healthcare centre	Both	Yes
Boulay P 04a, Short-term CR[36]	Canada	N = 37, 53.8 years (SD 9.9), 86.5% males	Exercise, education, information	N = 54, 6.5 years (SD 9.7), 77.8% males	12 months	Inpatient & Outpatient, Healthcare centre & University clinic,	Both	Unclear
Boulay P 04b, Long-term CR	Canada	N = 37, 54.3 years (SD 10.3), 78.4% males	Exercise, education, information	As above	As above	Inpatient & Outpatient, Healthcare centre & University clinic	Both	Unclear
Caliani S 04[39]	Spain	N = 113, 49.9 years (SD 8.4), Gender across groups 10 women	Exercise, education, reminders, dietary advice, psychological support, support group, relaxation	N = 40, 53.5 years (SD 9.5), Gender across groups 10 women.	3 & 12 months	Outpatient, Healthcare centre	Both	Yes
Coll-Fernandez R 14[37]	Spain	N = 521, 56 years (SD 10), 90% male	Exercise, smoking cessation, dietary modification, risk factor management, behaviour change intervention	N = 522, 67 years (SD 13), 71% male	18 months	Unclear	Unclear	Unclear
Junger C 10[41]	Germany	STEMI patients: N = 1649, Median age 63.2 years, 73.6% male. NSTEMI patients: N = 1107, Median age 66.3 years, 71.5% male	Exercise, risk factor management, education, counselling, psychological support, vocational support	STEMI patients: N = 783, Median age 70.0 years, 70% male. NSTEMI patients: N = 1008, Median age 71.3 years, 62.6% male	12 months	Inpatient, Specialist clinic	Group	Unclear
Kim C 11[42]	Korea	N = 69, 61.93 years (±10.67), 71% male	Exercise, risk factor management	N = 72, 64.49 (±9.31), 83% male	12 months	Unclear, Healthcare centre	Group	Unclear
Nielsen K 08[43]	Denmark	N = 145, 59.8 years, Gender N/R	Exercise, smoking cessation, dietary modification, healthcare professional consultation	N = 55, 59.7 years, Gender N/R.	12 & 24 months	Outpatient, Specialist clinic	Unclear	Yes
Rauch B 14[44]	Germany	N = 2513, 62 years (SD N/R), 76% male	Exercise, education, psychological support, vocational support	N = 1047, 69 years (SD N/R), 71% male	3 and 12 months	Outpatient, Specialist clinic	Unclear	Yes

SD Standard deviation, N/R Not reported

### Outcomes

#### All-cause mortality (Fig 3)

**Fig 3:**
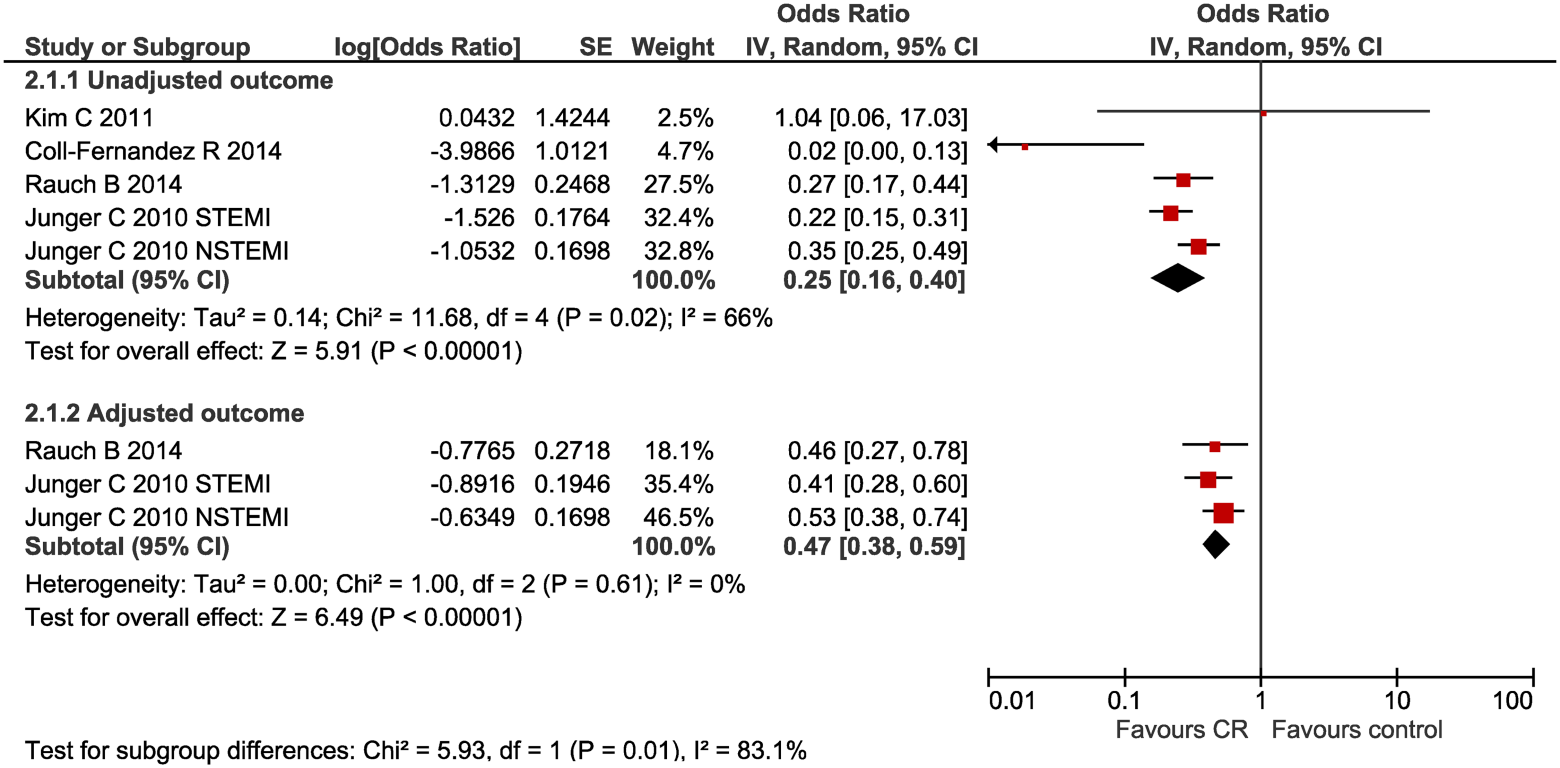
All-cause mortality forest plot.

Four studies assessed the impact of multi-component CR on all-cause mortality, two of which provided an adjusted outcome effect which could be synthesised. CR was related to a decreased risk of death from AMI; unadjusted OR 0.25 (95% CI 0.16,0.40) I^2^ = 66% and adjusted OR 0.47 (95% CI 0.38,0.59) I^2^ = 0%. One further study, which could not be synthesised with the adjusted ORs, reported an adjusted hazard ratio 0.08 (95% CI 0.01, 0.63) favouring CR [37].

#### Cardiac-related mortality (Fig 4)

**Fig 4:**
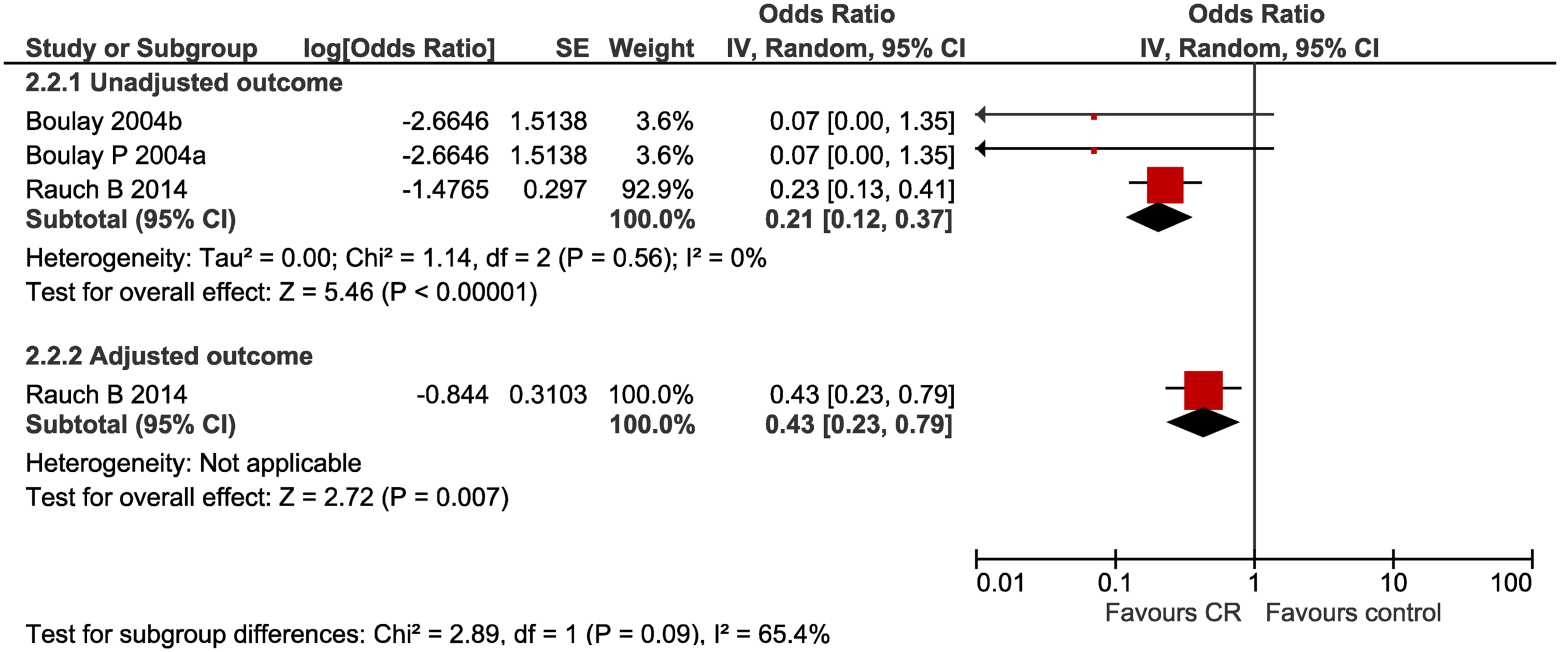
Cardiac-related mortality forest plot.

Two studies assessed the impact of multi-component CR on cardiac-related mortality, one of which provided an adjusted outcome effect. CR was related to a decreased risk of cardiac-related death from AMI; unadjusted OR 0.21 (95% CI 0.12, 0.37) I^2^ = 0% and adjusted OR 0.43 (95% CI 0.23, 0.79).

#### Hospital re-admission

Data could not be pooled from the two identified studies assessing the impact of multi-component CR on re-admission due to method of finding reporting. One study reported an adjusted effect, finding no significant effect from CR 0.96 (95% CI 0.81, 1.13).

#### Re-occurrence of MI (Fig 5)

**Fig 5:**
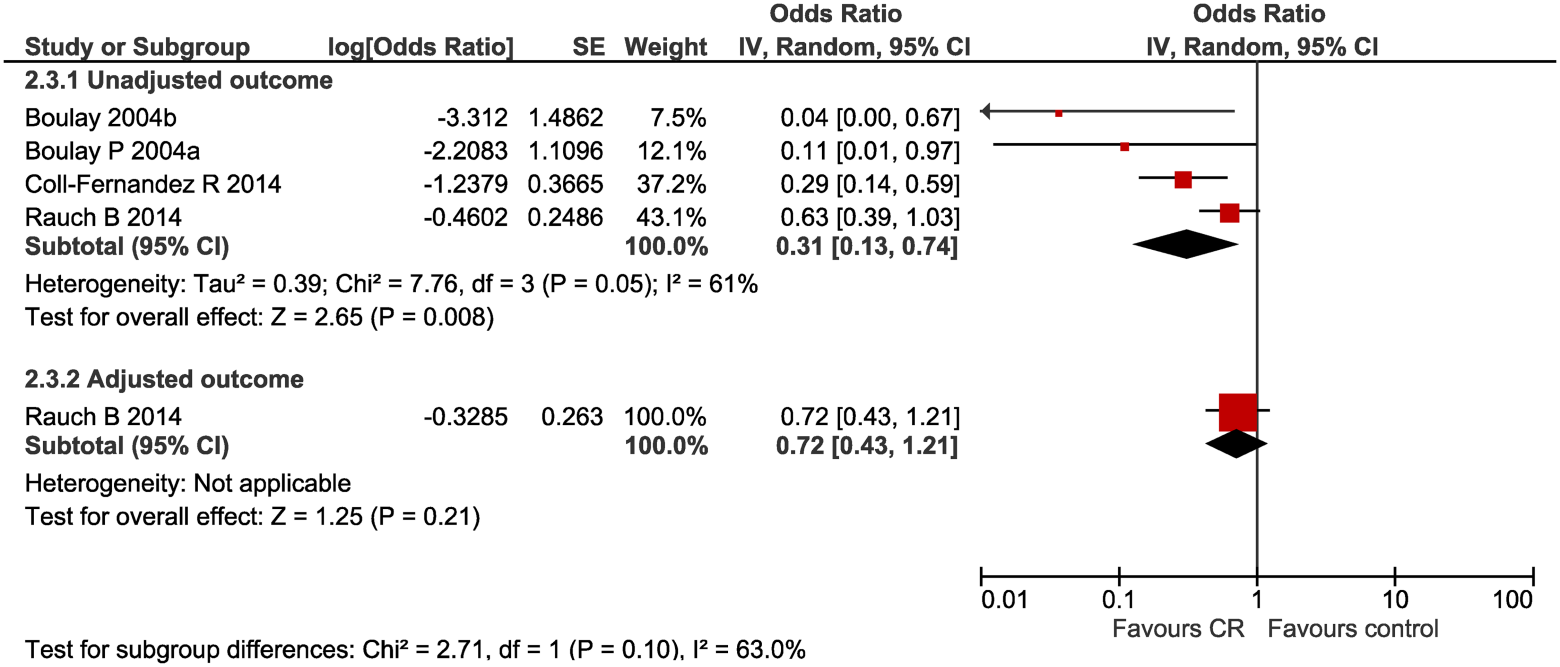
Re-occurrence of MI forest plot.

Three studies assessed the impact of multi-component CR on recurrent MI, one of which provided an adjusted outcome effect. CR was related to a decreased risk of recurrent MI in unadjusted analysis only; OR 0.31 (95% CI 0.13, 0.74) I^2^ = 61%. Adjusted analysis found no significant effect OR 0.72 (95% CI 0.43,1.21).

#### Re-vascularisation (Fig 6)

**Fig 6:**
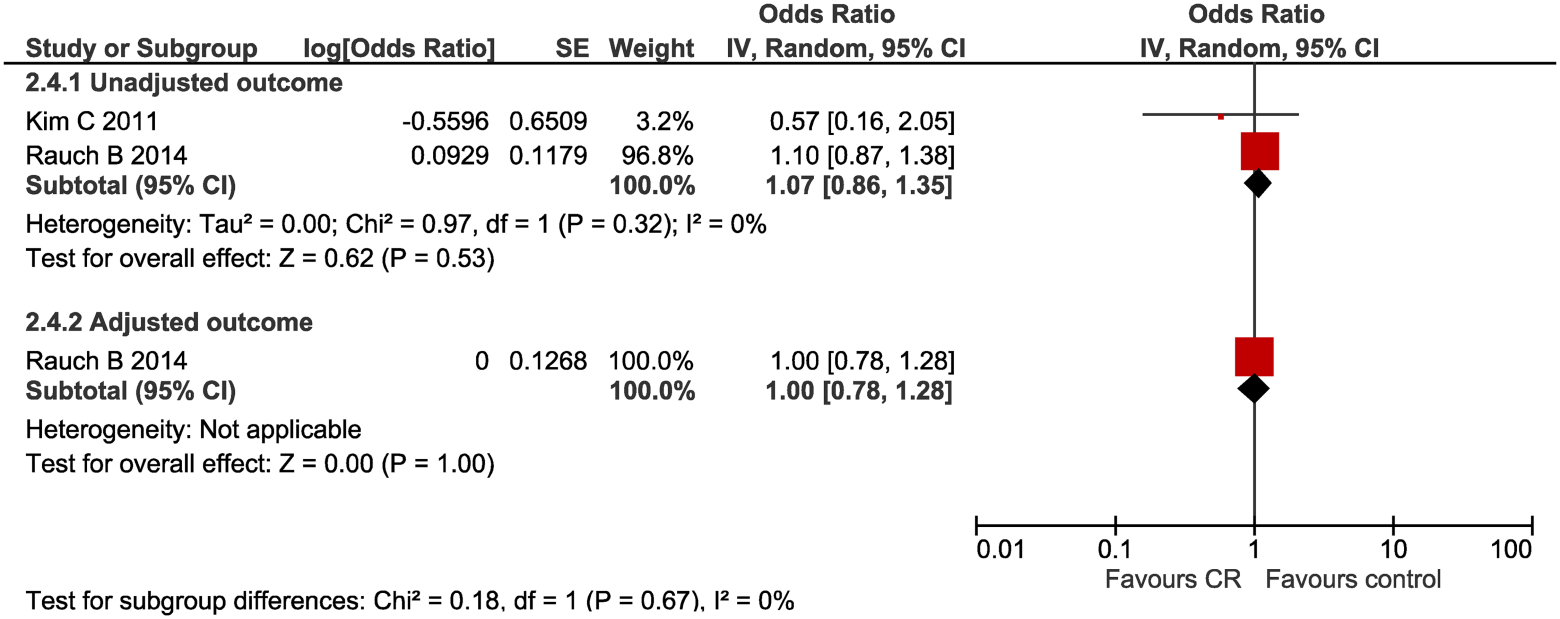
Re-vascularisation forest plot.

Two studies assessed the impact of multi-component CR on re-vascularisation following AMI, one of which provided an adjusted outcome effect. CR was not significantly related to a reduction in re-vascularisation in either unadjusted or adjusted effect measures; OR 1.07 (95% CI 0.86, 1.38) I^2^ = 0% and OR 1.00 (95% CI 0.78, 1.28) respectively.

#### HRQOL

Data could not be pooled from the two identified studies reporting HRQOL outcomes due to the heterogeneity of the outcome measures [38, 39]. Both studies reported significant improvements in quality of life in at least one domain at 6 months [38] and 1 year [39]. Neither study adjusted for confounding.

## Discussion

This study aimed to investigate routine CR in the context of modern cardiological practices. In addition it looked to extend the findings from the CROS review of observational CR data by exploring a homogenous patient sample (AMI only) and including HRQOL outcomes. A total of eight studies including 9836 AMI patients were eligible and were included in the analyses. Overall programmes reduced the risk of mortality, improved HRQOL but had no effect on re-vascularisation or re-hospitalisation. In an era where the existing RCT evidence base is aged, non-representative and the ethical challenges of conducting a new effectiveness trial when standard care is established as CR, this study has provided an important perspective on current day CR effectiveness in routine practice.

In comparison to the two most recent reviews of RCT evidence [9, 22] the findings from this study and the CROS study (a review of observational data in mixed CR participants) [21] drew differing conclusions to trial data. Specifically, opposite effects in total mortality and re-hospitalisation were found between observational and trial data, with a reduction in total mortality and no effect on re-hospitalisation found in observational studies. It may be argued that the observed differences may be due to the representativeness of trial evidence. Indeed the two recent trial based reviews of CR effectiveness [9, 22] include historical RCT trials which use exercise-only CR formats, include patients who had different care and treatment options historically versus modern day counter parts and the inherently different characteristics of RCT populations versus those receiving routine care. However, there were some similarities between trial and observational data; no reductions in recurrent MI were found and HRQOL improved. The positive effects on HRQOL found in AMI patient in this review are encouraging, however, as CROS did not consider HRQOL further work is needed to explore the effects in other CR populations.

With regard to the scope of evidence i.e. countries where evidence was available, only 8 studies conducted in 6 countries met the inclusion criteria. Observational data from other regions, particularly those with well-established CR programmes, would contribute substantially to a greater international perspective of current day CR performance, particularly in respect to alternative CR formats. Analysis of the programme characteristics did identify some differences between countries. Having a ‘multi-component’ CR programme formed part of the inclusion criteria for this review but a clear difference between American/ Canadian interventions and European equivalents was evident. That is, European programmes appeared to include many more components into their programmes. This difference may be driven by European standards stipulating a menu based approached to suit the needs of individual patients [4, 5]. Regardless, the impact of these differences requires investigation to identify the best approach and format [4] and greater utilisation of registry data could be one feasible route. Additionally all except one study, which did not report clearly, used a healthcare or clinic setting. Given the thriving research base on the alternative approaches for CR setting, such as home-based strategies [45, 46], it was surprising that no alternative settings were identified. To understand the impact of format and the use of different programme components greater access to registry data, which captures such information, would be invaluable.

### Limitations

No protocols were identified for the included studies, as such all quality assessment questions relating to pre-publication of a study protocol could not be assessed and were removed. There is a clear need for researchers of observational studies to pre-publish their study protocols. Many of the studies had small sample sizes and evidence of bias and thus the results from this review should be interpreted cautiously. In addition, only a few studies provided adjusted effect outcomes. Unadjusted outcomes are inherently bias, therefore adjusted and unadjusted outcomes were analysed separately and plotted alongside each other to permit comparison between studies where confounding had or had not been managed. Some heterogeneity was evident in the meta-analyses, but this did not exceed moderate levels and was thus appropriate to present graphically. Lastly due to insufficient data a sensitivity analysis on the impact of country of origin, study quality, and short versus long term follow-up was not possible as per the original review protocol. In addition, as we did not have access to individual patient level data we were unable to explore the impact of competing risk in our analyses.

## Conclusion

Current observational evidence; from both this review of AMI patients and the mixed CR populations in the CROS review, appear to contradict the most recent trial based reviews with respect to total mortality and re-hospitalisation. Arguably these differences highlight that analysis of data which is closer to clinical practice yields different findings to those found in clinical trials, which are known to recruit less representativeness populations. The usefulness of historic trial data in the modern cardiological era should also be questioned. Encouragingly however, the recent observational data shows CR reduces total mortality and improves HRQOL. Future work should seek to clarify which patient and service level factors determine the likelihood of achieving all-cause mortality, cardiac mortality or reduced re-admissions.

## Supporting information

S1 Table PRISMA checklist. (DOCX)

S2 Table Medline search strategy. (DOCX)
