# Supplementary material for: The effectiveness of modern cardiac rehabilitation: A systematic review of recent observational studies in non-attenders versus attenders
Source: PLoS One. 2017 May 12;12(5):e0177658. doi: 10.1371/journal.pone.0177658 (PMC5428953; doi:10.1371/journal.pone.0177658)
Supplement: S2 Table — (DOCX) [file pone.0177658.s002.docx]

| **Searches** | **Ovid MEDLINE(R) In-Process & Other Non-Indexed Citations and**  **Ovid MEDLINE(R)** 1946 to Present. |
| --- | --- |
| 1 | exp myocardial infarction/ |
| 2 | myocardial ischemia/ |
| 3 | coronary disease/ |
| 4 | heart disease/ |
| 5 | (myocard$5 adj3 (infarct$ or ischemia$ or revasc$ or disease$)).ti,ab. |
| 6 | ((ischaemi$2 or ischemi$2) adj3 heart).ti,ab. |
| 7 | (infarct$5 adj5 heart).ti,ab. |
| 8 | myocardial revascularization/ |
| 9 | coronary artery bypass.ab,ti. |
| 10 | CABG.ti,ab. |
| 11 | PTCA.ti,ab. |
| 12 | ((heart or myocard$5 or coronary or cardiac) adj stent$).ti,ab. |
| 13 | heart bypass, left/ |
| 14 | heart bypass, right/ |
| 15 | 1 or 2 or 3 or 4 or 5 or 6 or 7 or 8 or 9 or 10 or 11 or 12 or 13 or 14 |
| 16 | rehabilitation/ |
| 17 | rehabilitation centers/ |
| 18 | rehabilitat$.ti,ab. |
| 19 | secondary prevention/ |
| 20 | exercise therapy/ |

| 21 | physical exertion/ |
| --- | --- |
| 22 | (physical$4 adj (fit or fitness or train$5 or therap$5 or activit$5 or exercis$5)).ti,ab. |
| 23 | ((exercis$5 or fitness) adj (treatment or intervent$4 or program$2 or therapy)).ti,ab. |
| 24 | Patient Education as Topic/ |
| 25 | ((patient$ or health) adj (education or promot$)).ti,ab. |
| 26 | health education/ |
| 27 | health promotion/ |
| 28 | ((lifestyle or life-style) adj (treatment or intervent$4 or program$2 or therapy)).ti,ab. |
| 29 | self care/ |
| 30 | (self adj (manage$5 or care or motivate$5)).ti,ab. |
| 31 | counseling/ |
| 32 | counsel$.ti,ab. |
| 33 | psychotherapy/ |
| 34 | psychotherap$3.ti,ab. |
| 35 | health behavior/ |
| 36 | (behavio$ adj (modify or modificat$ or therap$ or change)).ti,ab. |
| 37 | 16 or 17 or 18 or 19 or 20 or 21 or 22 or 23 or 24 or 25 or 26 or 27 or 28 or 29 or 30 or  31 or 32 or 33 or 34 or 35 or 36 |
| 38 | participat$.ti,ab. |
| 39 | attend$.ti,ab. |
| 40 | (Referral and Consultation).mp. [mp=title, abstract, original title, name of substance word, subject heading word, keyword heading word, protocol supplementary concept |

|  | word, rare disease supplementary concept word, unique identifier] |
| --- | --- |
| 41 | uptake.ti,ab. |
| 42 | take up.ti,ab. |
| 43 | taking up.ti,ab. |
| 44 | user$.ti,ab. |
| 45 | (nonuse$ or non-use$).ti,ab. |
| 46 | (nonparticipat$ or non- participat$).ti,ab. |
| 47 | (nonattend$ or non-attend$).ti,ab. |
| 48 | (utiliz$ or utilis$).ti,ab. |
| 49 | (non-utiliz$ or non-utilis$).ti,ab. |
| 50 | 38 or 39 or 40 or 41 or 42 or 43 or 44 or 45 or 46 or 47 or 48 or 49 |
| 51 | prognosis/ |
| 52 | prognosis.ti,ab. |
| 53 | mortality/ |
| 54 | mortality.ti,ab. |
| 55 | patient readmission/ |
| 56 | readmi$5.ti,ab. |
| 57 | epidemiology/ |
| 58 | epidemiology.ti,ab. |
| 59 | treatment outcome/ |
| 60 | (treatment adj outcome).ti,ab. |
| 61 | 51 or 52 or 53 or 54 or 55 or 56 or 57 or 58 or 59 or 60 |
| 62 | (cardiac rehabilitat$ adj User$).ti,ab. |
| 63 | (cardiac rehabilitat$ adj attend$).ti,ab. |
| 64 | (cardiac rehabilitat$ adj participat$).ti,ab. |
| 65 | 62 or 63 or 64 |
| 66 | exp animals/ not humans.sh. |
| 67 | 15 and 37 |
| 68 | 67 and 50 |
| 69 | 68 and 61 |
| 70 | 69 or 65 |
| **71** | **70 not 66** |
